# m6A Ribonucleic Acid Methylation in Fibrotic Diseases of Visceral Organs

**DOI:** 10.1002/smsc.202400308

**Published:** 2024-11-21

**Authors:** Xiaoniu Dai, Yusi Cheng, Wei Luo, Jing Wang, Cuifen Wang, Xinxin Zhang, Wei Zhang, Jie Chao

**Affiliations:** ^1^ Jiangsu Provincial Key Laboratory of Critical Care Medicine Department of Physiology, School of Medicine Zhongda Hospital Southeast University 87 Dingjiaqiao Rd Nanjing Jiangsu 210009 China; ^2^ Tissue Sciences Facility University of Nebraska Medical Center 985815 Nebraska Medical Center Omaha NE6B19B‐5815 USA

**Keywords:** cardiac fibrosis, hepatic fibrosis, N6‐methyladenosine, pulmonary fibrosis renal fibrosis

## Abstract

Fibrosis is a pathological process characterized by the excessive deposition of extracellular matrix in the tissue's extracellular space, leading to structural injury and organ dysfunction, and even organ failure, posing a threat to human life. Despite mounting evidence suggesting that fibrosis is reversible, effective treatments for fibrotic diseases are lacking. Accumulating evidence has elucidated that ribonucleic acid (RNA) modifications have emerged as novel mechanisms regulating gene expression. N6‐methyladenosine (m6A) modification is a well‐known prevalent RNA posttranscriptional modification that participates in essential biological processes such as RNA splicing, translation, and degradation. It is tightly implicated in a wide range of cellular processes and various human diseases, particularly in organ fibrosis. The m6A modification is a dynamic and reversible process regulated by methylases, commonly known as “writers,” and demethylases referred to as “erasers,” while m6A modifications are recognized by “readers.” Accumulating evidence suggests that m6A modification on RNAs is tightly associated with fibrotic diseases of visceral organs including the lungs, heart, liver, and kidney. In this review, recent advances in the impact of m6A methylation of RNAs on visceral organ fibrosis are highlighted and the potential prospects for therapy in treating fibrotic diseases of visceral organs are discussed.

## Graphical abstract

This review provides a systematic overview of recent studies on m6A modification in fibrotic diseases affecting visceral organs, including the lungs, heart, liver, and kidneys. Furthermore, it emphasizes the significant advancements in understanding how m6A methylation of ribonucleic acid influences fibrosis in these organs and explores potential therapeutic avenues for treating fibrotic diseases affecting visceral structures.

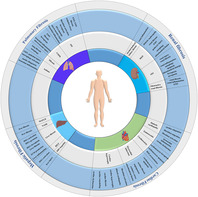

## 1 Introduction

Fibrosis is a significant contributor to global morbidity and mortality. The common pathological process of fibrosis is characterized by the excessive deposition of extracellular matrix (ECM), leading to the gradual replacement of normal tissue structure by fibrotic tissue in specific tissues and organs.^[^ ^1^ ^]^ Tissue homeostasis in the microenvironment is regulated by various cell populations within the tissues. During the development of fibrosis, persistent and repetitive damage leads to excessive repair processes, with activation of fibroblasts and significant deposition of collagen molecules. Additionally, injury signals mediated by epithelial and endothelial cells, as well as mesenchymal transition, promote fibrogenesis. Macrophage‐initiated immune and inflammatory responses also play a role in this process. Activated resident macrophages and monocyte‐derived macrophages secrete TGF‐β to trigger innate immunity. Increased permeability of endothelial cells allows circulating immune cells to approach fibroblasts.^[^ ^2^ ^]^ Fibrosis can affect various tissues and organs, including the lung, heart, liver, and kidney.^[^ ^3^ ^]^ While preclinical and clinical studies have shown that fibrosis is reversible, unfortunately there are still limited clinical options for treating visceral organ fibrosis. Therefore, it is crucial to explore the mechanisms of fibrosis progression and develop specific drugs that can reverse this condition.

**Figure 1:**
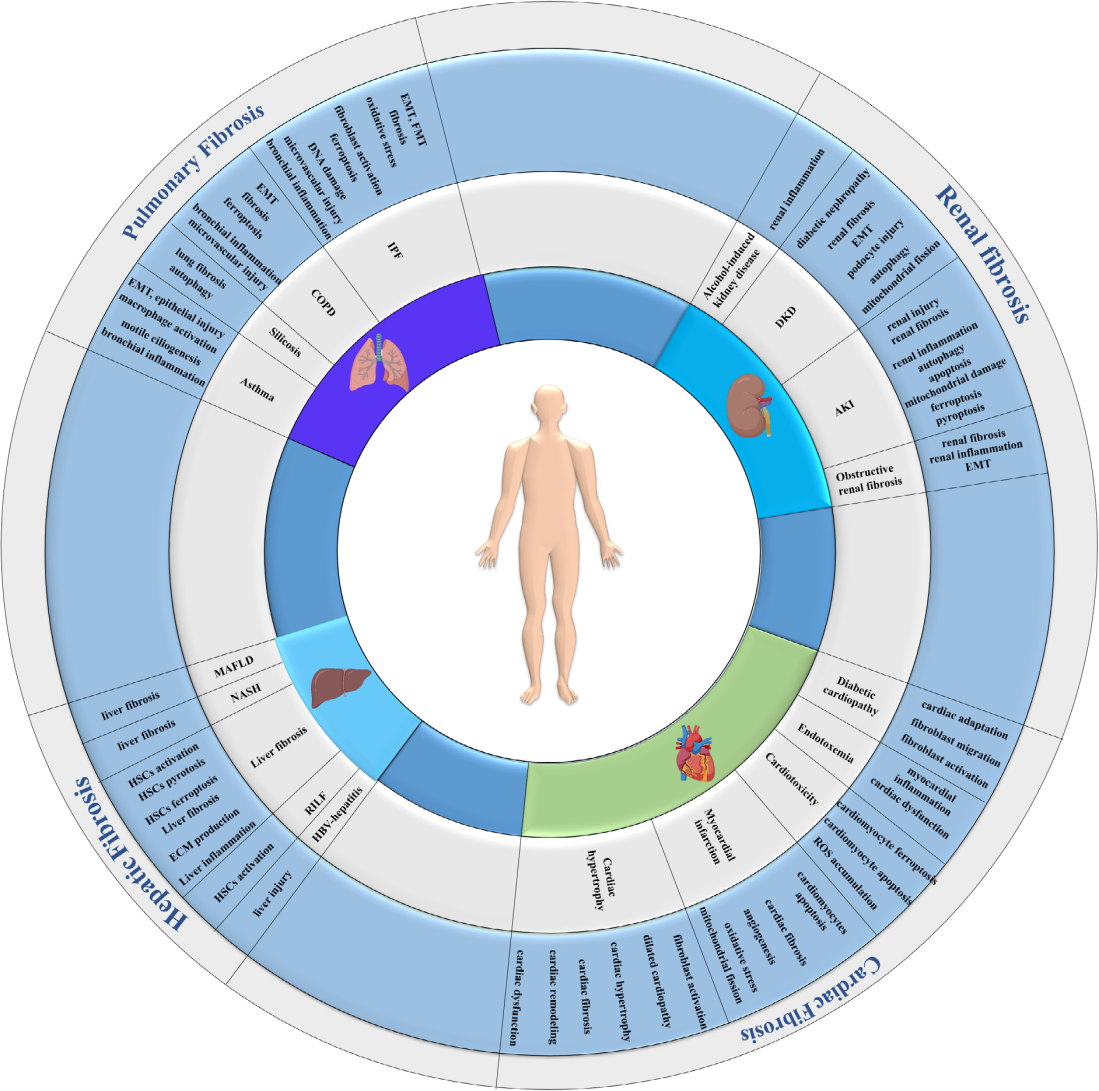
An overview of the functional roles of m6A modification in fibrotic diseases affecting the lung, heart, liver, and kidney.

N6‐methyladenosine (m6A) methylation was first identified in mammalian mRNA in 1974. However, technical limitations hindered the systematic analysis of its occurrence and functions, leading to a stagnation in research on m6A modification.^[^ ^4^ ^]^ It was not until the last decade that sufficient sensitivity and high‐resolution genome‐wide techniques were developed to detect and quantify these modifications in low‐abundance RNA species.^[^ ^5^, ^6^ ^]^ Recent advances in high‐throughput sequencing technology, combined with selective chemical and immunological identification of modified nucleotides, have accelerated the mapping of modifications on abundant tRNAs and rRNAs as well as less‐abundant mRNAs from prokaryotic bacteria to eukaryotic human beings.^[^ ^7^, ^8^ ^]^ Accumulating reports have confirmed that m6A mRNA modifications are linked to regulation at multiple steps in mRNA processing, establishing m6A methylation on mRNA as a research hotspot.^[^ ^6^ ^]^ Numerous studies focusing on m6A RNA methylation have documented its significant impact on various mammalian biological processes including meiotic progression, deoxyribonucleic acid (DNA) damage response, cell cycle regulation, heat shock response, circadian rhythm control, myogenesis, fat differentiation, and development of hematopoietic system as well as central nervous and reproductive systems.^[^ ^6^, ^9^, ^10^, ^11^ ^]^ m6A is a common mRNA modification with important biological functions, playing a role in post‐transcriptional gene regulation. It has also been identified as a potential diagnostic marker and therapeutic target for various diseases. This review summarizes the mechanisms of m6A RNA methylation, its regulators, and downstream target genes/proteins in organ fibrosis (**Table** **1** and **Figure** **1**), focusing on lung, heart, liver, and kidney. It also discusses the potential of epigenetic therapy for treating fibrotic diseases affecting visceral organs.

**Table 1:** List of m6A RNA methylation in fibrotic diseases of visceral organs.

Organ fibrosis	Diseases	Regulator	Function	Mechanism	Sample	References
Pulmonary fibrosis	IPF	METTL3↑	Promoting FMT process	Promoting KCNQ6 expression	C57BL/6J mice, WI‐38 and HEK293T cells	[31]
		METTL3↑	Promoting Epithelial‐mesenchymal transition (EMT) progression	Promoting CDH1 mRNA stability	C57BL/6 male mice, human normal bronchial epithelium cell line BEAS‐2B	[33]
		METTL3↑	Protection against oxidative stress	Promoting Nrf2 translation	male C57BL/6 mice, HBEC line (16HBE)	[34]
		YTHDF1↑	Promoting FMT process	Promoting NREP expression	male C57BL/6J mice, MLE12 cells	[35]
		YTHDC1↓	Facilitating DNA damage repair	Activation of ATR kinase	HEK293T, A549 and L2 cells	[36]
	COPD	METTL16↑	Promoting microvascular injury	Inhibiting Sulf2 expression	platelet‐poor plasma from patients with COPD, male Sprague‐Dawley rats	[37]
		METTL3↑	Promoting EMT progression	Inhibiting SOCS3 expression	HBEC line (16HBE)	[41]
		YTHDF1↑	Triggering CSE‐induced ferroptosis	Promoting IREB2 translation	lung of COPD smokers, male BALB/c mice, HBECs, human pulmonary alveolar epithelial cells (HPAEpiCs),	[42]
		ZC3H13↑	Promoting inflammation and fibrosis of bronchial epithelial cell	Promoting ITGA6 mRNA expression and stability	male C57BL/6 mice, HBECs BEAS‐2B, human bronchial epithelial‐like cells 16HBE	[43]
	Silicosis	METTL3↑	Promoting pulmonary fibrosis	Promoting circRNAs binding to eIF4A3	lung tissue of mice and human pulmonary fibroblasts treated with SiO2	[47]
		ALKBH5↓	Inhibiting macrophage autophagy	Promoting Slamf7 mRNA expression and stability	male C57BL/6 mice, RAW264.7 and THP‐1 cells	[48]
		ALKBH5↓	Promoting silica‐induced lung fibrosis	Inhibiting export of ZNF609	male C57BL/6 mice, MRC‐5 cells	[49]
		ALKBH5↑	Promoting silica‐induced lung fibrosis	Inhibiting FOXM1 expression	male C57BL/6 mice, MRC‐5 cells	[50]
	Asthma	METTL3↓	Promoting EMT in 16HBE cells Promoting ECM production in HPF cells	Inhibiting Smad3 expression	alveolar lavage fluid samples collected from asthmatic patients, 16HBE and HPF cells	[55]
		METTL3↓	Promoting M2 macrophage activation	Promoting PTX3 mRNA expression and stability	human PBMCs, human monocyte‐derived macrophages, C57BL/6 mice	[56]
		METTL3↓	Inhibiting Th2 cell differentiation	Promoting SOX5 expression	peripheral blood mononuclear cells (PBMCs), C57BL/6 female mice, adults diagnosed with T2 asthma	[57]
		METTL3↓	Stimulation of ASMCs	Promoting HBD‐3 expression	venous blood from children with asthma, human ASMCs	[58]
		METTL3↑	Promoting DNA damage in PM2.5‐treated HBE cells	Inhibiting COX4I1 expression	HBE cells	[59]
		FTO↑	Promoting motile ciliogenesis	Promoting FOXJ1 expression	C57BL/6J mice, Lenti‐X 293T, HCT‐8, HeLa, SW‐480 and Capan‐2 cells	[60]
		FTO↑	Exacerbating impairment of the epithelial barrier	Promoting IKBKB mRNA expression and stability	C57BL6 mice, Beas‐2B cells	[61]
		YTHDF1↑	Promoting NLRP3 inflammasome production and IL‐β secretion in AECs cell	Promoting CLOCK expression	C57/BL6 mice and BALB/c mice, human bronchial biopsies samples from patients with asthma	[63]
Cardiac fibrosis	Cardiac hypertrophy	METTL3↑	Promoting CF activation and cardiac fibrosis Promoting atrial fibrillation	Promoting IGFBP3 expression	atrial tissues of patients with atrial fibrillation, CFs, C57/BL6 mice	[71]
		WTAP↓	Inducing dilated cardiomyopathy	Promoting Mef2c expression	mice, primary rat or mouse cardiomyocytes	[83]
		FTO↓	Reducing ejection fraction and fractional shortening Increasing degree of dilatation increases	Changing m6A methylation in metabolic and regulatory signal pathway	heart tissue from mice and patients with heart failure,	[84]
		YTHDF2↓	Alleviating myocardial hypertrophy	Inhibiting expression of Myh7 and Eef2	C57BL/6J mice, human left ventricular samples with heart failure	[98]
		YTHDF2↓	Promoting pathological cardiac remodeling and dysfunction	Promoting MYZAP expression	adult male and female C57BL/6N mice, mouse cardiomyocytes	[100, 101]
		YTHDF2↑	Promoting cardiac hypertrophy	Promoting CPT‐1a degradation	male C57BL/6J mice, primary culture of neonatal rat ventricular myocytes (NRVMs)	[102]
		YTHDC1↓	Inducing dilated cardiomyopathy	Promoting Titin expression	cardiomyocytes and heart tissue of C57BL/6J mice,	[104]
		IGF2BP2↑	Repressing cardiac hypertrophy	Collaborating with miR‐133a and AGO2	Cardiomyocytes and heart tissue of transgenic mice (Jackson Labs)	[105, 106]
	Myocardial infarction	WTAP↓	Inhibiting oxidative stress and preserving cardiac function	Inhibiting TXNIP expression	male C57BL/6J mice, H9C2 cells	[65]
		METTL3↑	Promoting cardiac fibrosis	Promoting pro‐fibrosis factors production	male C57BL/6N mice, CFs	[69]
		METTL3↑	Promoting CF proliferation and FMT progression	Promoting Smad2/3 expression	male C57BL/6N mice, CFs	[70]
		METTL3↑	Promoting cardiomyocyte pyroptosis	Inhibiting PRKCE mRNA expression	myocardial tissues and blood of male Sprague‐Dawley rats	[73]
		METTL3↓	Promoting cardiomyocytes regeneration and cardiac repair	Promoting Yap/Ctnnd1 expression	old male C57BL/6 mice, neonatal ventricular myocytes of rat and human AC‐16 cell	[72]
		METTL3↑	Improving cardiomyocyte proliferation and ameliorating MI	Downregulating miR‐17‐3p expression	heart samples of patients with MI, male C57BL6/J mice and Sprague‐Dawley rats	[74]
		METTL3↑	Improving cardiac regeneration and function	Suppressing PSPH expression and inhibiting activity of CDK2	C57BL/6 mice, cardiomyocytes	[75]
		METTL3↑	Promoting mitochondrial fission and cardiac fibrosis	Promoting DNA‐PKcs activity	transgenic mice (Jackson Labs), HL‐1 cells	[79]
		METTL3↑, ALKBH5↓	Inhibiting autophagic flux and promoting apoptosis in cardiomyocytes	Inhibiting TFEB expression	H9c2 cardiomyocytes, HEK293T cells, C57BL6/J mouse	[80]
		METTL3↑	Inhibiting cardiomyocyte proliferation and heart regeneration	Inhibiting Fgf16 expression	rat cardiomyocytes, male and female C57BL/6J mice	[81]
		METTL3↑	Promoting cardiac fibrosis and cardiomyocyte apoptosis	Promoting TNC mRNA stability	C57/BL mice, HL1 and AC16 cells	[82]
		FTO↓	Reducing fibrosis and enhanced angiogenesis.	Reducing SERCA2a expression	human left ventricular tissues, male and female mice (c57Bl6) and Sprague‐Dawley rats	[85]
		ALKBH5↑	Impairing endothelial cell angiogenesis	Inhibiting SPHK1 expression	human umbilical vein endothelial cells (HUVEC) and human microvascular endothelial cells (HMVE)	[86]
		ALKBH5↓	Inducing senescent cardiomyocytes apoptosis	Inhibiting STAT3 expression	Sprague‐Dawley rats, H9c2 and HEK293A cells	[87]
		ALKBH5↑	Reducing infarct size and ameliorating cardiac function	Promoting ErbB4 expression	male C57BL/6 mice, cardiomyocytes and fibroblasts from neonatal mice	[88]
		FTO↓	Promoting cardiomyocytes apoptosis	Inhibiting Mhrt expression	male C57BL/6 mice, mouse myocardial cells	[89]
		FTO↓	Promoting viability, activation and migration of cardiomyocytes	Promoting DKK2 expression	primary human CFs, C57BL/6 mice	[92]
		hnRNPC↑	Promoting pathological remodeling of ECM	Relocating itself to the sarcomeric Z‐disc	cardiac specimen from patients with heart failure, C57BL/6 mice, human iPSC line DF 19‐9‐7T (WiCell) and NHDFs	[107]
	Diabetic cardiopathy	WTAP↑ YTHDF2↑	Inducing activation, proliferation and migration of CF	Promoting Decr1 expression	CFs, leptin receptor‐deficient mice, human myocardial tissue samples	[113]
		FTO↑ ALKBH5↑	Promoting cardiac adaptation to fasting	Regulating Nox4 and Hdac1 transcription	male Wistar rats, AVCMs	[114]
	Cardiotoxicity	FTO↓	Promoting DOX‐induced ferroptosis	Inhibiting P21/Nrf2 activation	C57BL/6J mice, H9C2 cells	[93]
		FTO↑	Attenuating cardiomyocyte apoptosis, reducing ROS accumulation and ameliorating the mitochondrial iron overload	circ‐ZNF609 knockdown enhancing FTO expression	adult male C57BL/6J mice, primary neonatal rat cardiomyocyte	[94]
		METTL3↑	Promoting cardiomyocyte ferroptosis	Inhibiting TFRC expression	C57BL/6J mice, adult mouse cardiomyocytes (AMCMs), myocardial samples	[95]
	Endotoxemia	FTO↓	Promoting myocardial inflammation and cardiac dysfunction	Promoting IL‐6 and TNF‐α translation	C57BL/6J mice, cardiomyoblasts, H9c2 cells	[96]
Hepatic fibrosis	Liver fibrosis	METTL3↑	Activating HSCs	Inhibiting SPRY2 expression	C57BL/6J mice, HSC‐T6 cells	[122]
		METTL3↑	Activating HSCs	Promoting Lats2 expression	C57BL/6 mice, HSCs isolated from mice	[123]
		METTL3↑	Stimulating pyroptosis and inflammation of macrophages	Promoting TAK1 expression	C57/BL6 male mice, Kupffer, HEK293T and RAW264.7 cells	[124]
		METTL14↓	Activating HSCs	Promoting NOVA2 expression	LX2 cell, male C57BL/6J mice	[126]
		YTHDF1↑ FTO↓	Inducing HSC ferroptosis	Promoting BECN1 stability and expression	primary HSCs from human liver tissue and from ICR mice, ICR mice	[129, 130]
		ALKBH5↓	Activating HSCs	Inhibiting PTCH1 expression	male C57BL/6 mice, HSCs	[131]
		WTAP↑	Inducing liver fibrosis	Promoting Ptch1 expression	Male Sprague‐Dawley rats, HSCs	[133]
		YTHDC1↑	Promoting liver fibrosis	Promoting NR1D1 mRNA degradation	male ICR mice, HSCs, hepatocytes, Kupffer cells	[134]
		YTHDF1↑	Promoting ECM production	Promoting collagens mRNA stability	JS1 cells, C57BL/6 mice	[135]
		YTHDF2↑	Relieving liver fibrosis	Promoting FOXO3 expression	human liver specimens, C57BL/6J mice, LX‐2 cells,	[136]
		YTHDF3↑	Promoting ECM production	Promoting PRDX3 expression	human fibrotic liver tissue, male C57BL/6 mice, LX‐2 cell and mouse primary HSCs	[137]
		IGF2BP2↑	Activating HSCs	Promoting ALDOA expression	human liver samples, male C57BL/6 mice, LX‐2 cells	[138]
	NASH	METTL3/14↑	Promoting NASH to liver fibrosis	Promoting TGF‐β1 expression	male Sprague Dawley rats, C57BL/6J mice, primary Kupffer Cells	[121]
	MAFLD	METTL14↓	Promoting liver fibrosis	Downregulating GLS2 expression	liver tissues from patients with MAFLD, mice	[127]
	RILF	ALKBH5↑	Activating HSCs	Promoting TIRAP expression	male C57BL/6 mice, hepatocellular carcinoma samples from patients with radiotherapy	[132]
	HBV‐hepatitis	METTL3↑	Promoting liver injury	Promoting maturation of miR‐146a‐5p	C57BL/6 male mice, human hepatic cells THLE‐2	[125]
Renal fibrosis	Obstructive renal fibrosis	METTL3↑	Promoting kidney fibrosis	Promoting NET1 expression	HK‐2 cells, UUO mouse model	[145]
		METTL3↑	Promoting renal inflammation and injury	Promoting SPRY1 expression	UUO model in C57BL/6 mice, HK‐2 cells	[148]
		METTL3↑	Promoting renal fibrosis	Promoting FAK expression	HK2 cells, kidney tissue from patients with renal fibrosis, UUO model in C57BL/6 mice	[149]
		METTL3↑	Promoting renal fibrosis	Promoting CTGF expression	mouse glomerular mesangial cell line SV40 MES13, UUO model in C57BL/6 mice	[151]
		FTO↑	Promoting renal fibrosis	Promoting RUNX1 expression	TECs, UUO model in C57BL/6 mice	[153]
		FTO↓	Promoting renal fibrosis	Promoting TASK‐2 expression	HK‐2 cells, UUO model in C57BL/6 mice	[155]
		FTO↑	Promoting EMT process and inflammation response	Inhibiting lncRNA GAS5 expression	HK‐2 cells, UUO model in C57BL/6 mice	[157]
		ALKBH5↓	Inhibiting renal fibrosis	Reducing Snail expression	HK‐2 cells, UUO model in C57BL/6 mice	[159]
		YTHDF1↑	Promoting renal fibrosis	Promoting YAP1 expression	human fibrotic kidneys, UUO model in C57BL/6 mice	[164]
	AKI	METTL3↑	Promoting renal inflammation and injury	Enhancing TAB3 expression	Human kidney TEC line (HK‐2) and mTECs, kidney tissue from patients with AKI, Lipopolysaccharide (LPS)‐induced AKI in C57BL/6 mice	[147]
		METTL3↑	Promoting renal injury and cell apoptosis	Promoting FOXD1 expression	IRI‐induced AKI model in Sprague‐Dawley rats, NRK‐52E cells	[150]
		METTL14↑	Promoting renal injury	Inhibiting YAP1 expression	IRI‐induced AKI model in C57BL/6 mice, HK‐2 cells	[152]
		FTO↓	Promoting renal injury	Inhibiting AQP3 expression	human proximal TECs (PTECs) HK‐2	[154]
		FTO↓	Inhibiting autophagy	Promoting ATG7 expression	kidney tissue from patients with S‐AKI, HK‐2 cells, S‐AKI model in C57BL/6 mice	[158]
		ALKBH5↓	Promoting renal fibrosis	Increasing CCL28 expression	mouse renal TECs (mRTECs), I/R‐induced renal injury in C57BL/6 mice	[161]
		WTAP↑	Promoting renal inflammation, mitochondrial damage and ferroptosis	Promoting LMNB1 expression	AKI mice model with CLP, HK2 cells	[162]
		IGF2BP1↑	Promoting renal pyroptosis	Promoting E2F1 expression	kidney tissue from patients with AKI, HK2 cells	[163]
	Kidney fibrosis	METTL3↑	Promoting kidney fibrosis	Promoting EVL mRNA stability and expression	IRI‐induced AKI model in C57BL/6 mice, HK2 cells	[146]
	Alcohol‐induced kidney injury	FTO↓	Promoting renal inflammation	Inhibiting PPAR‐α expression	alcoholic kidney injury in male C57BL/6 mice, HK2 cells	[156]
	DKD	METTL3↑	Promoting DN	Promoting PINK1 expression	HK2 cells, high‐fat and high‐sugar diet induced DKD	[166]
		METTL3↓	Promoting DN	Promoting NSD2 expression	kidney tissue from patients with DN, HFD induced DN in male C57BL/6 mice, mouse mesangial cell line	[167]
		METTL3↑	Promoting podocyte injury in DN	Promoting TIMP2 expression	kidney tissue from patients with DM, male C57BL/6J mice, mouse podocytes (MPC5)	[168]
		METTL3↑	Promoting macroautophagy/autophagy in kidney	Inhibiting Mir665‐3p‐Atg4b expression	kidney tissue patients with DN, mouse podocytes (MPC5)	[169]
		METTL14↑	Promoting glomerular endothelial cell injury	Promoting α‐klotho expression	renal samples of DN patients, human renal glomerular endothelial cells (HRGECs)	[170]
		METTL14↑	Promoting EMT process	Inhibiting PTEN expression	DM and UUO model in C57BL/6J mice, HK2 cells	[171]
		METTL14↑	Promoting podocyte injury	Promoting Sirt1 expression	DM model in C57BL/6 mice, kidney samples from DM patients, human podocytes	[172]
		FTO↑	Promoting podocyte injury and inflammation	Promoting SAA2 expression	human podocytes (HPC), mouse primary podocytes, C57BL/6 mice	[173]
		FTO↓	Promoting kidney inflammation	Reducing SOCS1 expression	kidney samples from DM patients	[174]
		FTO↑	Prompting podocyte injury	Inhibiting RAB3B expression	kidney samples from DM patients, human podocytes (HPCs)	[175]
		FTO↑	Promoting autophagy	Promoting SQSTM1 expression	I/R and UUO model in C57BL/6 mice, HK2 cells	[176]
		IGF2BP2↑	Promoting macrophage infiltration and renal fibrosis	Promoting SP1 expression	HK2 cells, DKD model in C57BL/BKS mice	[177]
		IGF2BP3↓	Inhibiting mitochondrial fission and repressing cell apoptosis	promoting CAMK1 expression	kidney samples from DN patients, male C57BL/6 mice, HK2 cells	[178]

## 2 Overview of m6A Modification

In recent years, the most extensively studied epitranscriptomic mark on RNA is m6A methylation modification, which occurs in messenger RNAs (mRNA) and most noncoding RNAs (ncRNAs), including rRNAs, tRNAs, miRNAs, circRNAs, snRNAs, snoRNAs, and lncRNAs.^[^ ^6^, ^12^, ^13^ ^]^ The reversible process of m6A methylation is regulated by diverse methylases and demethylases and plays a crucial role in regulating both physiological and pathological processes of mRNA.^[^ ^14^ ^]^

**Figure 2:**
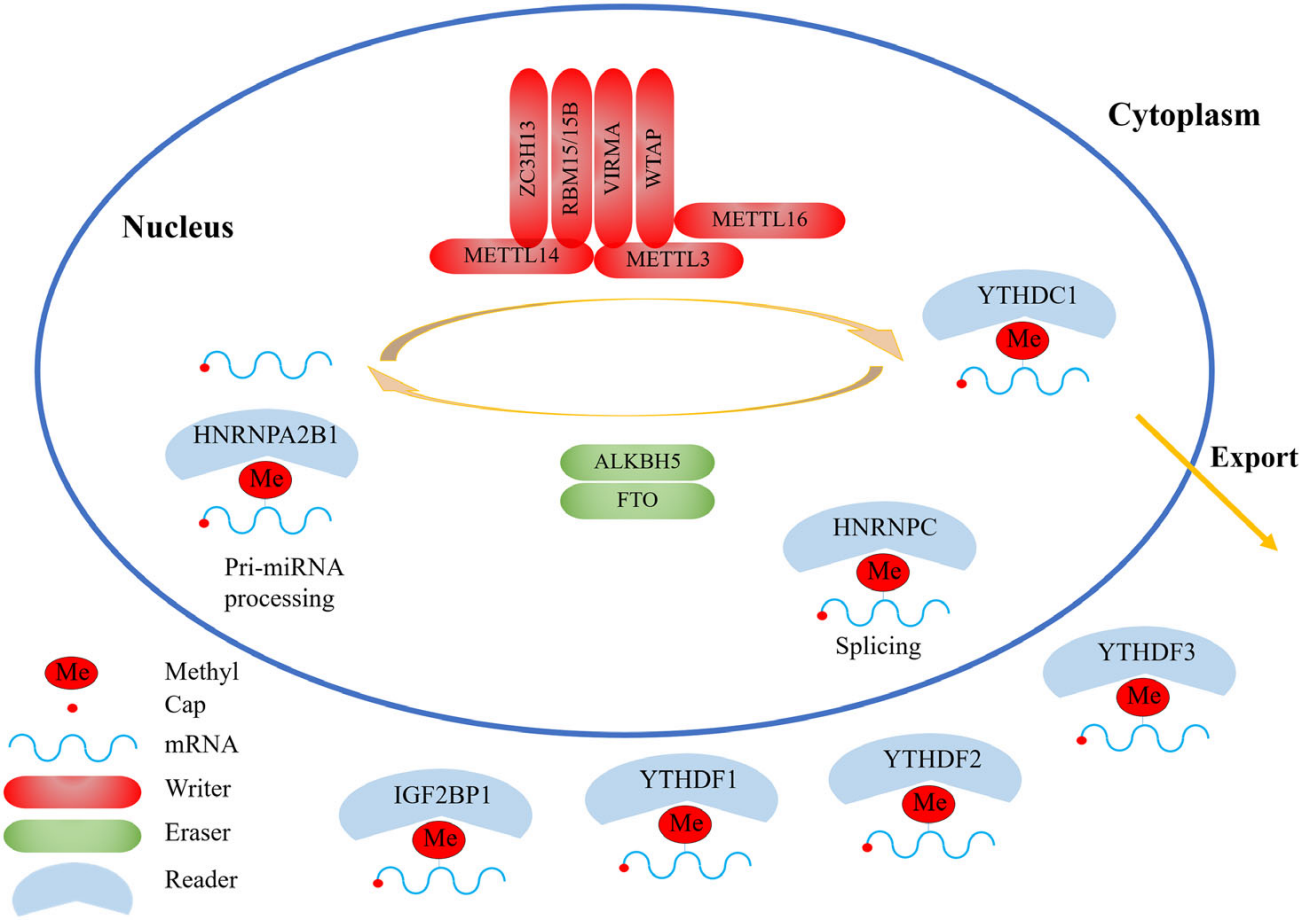
The dynamic and reversible processes and consequences of m6A methylation.

The regulators of m6A modification can be categorized into three groups: methyltransferases (writers), demethylases (erasers), and binding proteins (readers), as shown in **Figure** **2**.^[^ ^15^ ^]^ RNA is methylated by the m6A writer and demethylated by the m6A eraser. N6‐methylated RNA is recognized and bound by a specific m6A reader, enabling the regulation of gene expression.^[^ ^16^ ^]^ Methyltransferases, including METTL3, METTL14, along with adapter proteins WTAP, METTL16, RBM15/15B, ZC3H13, and VIRMA (also known as KIAA1429), assemble into protein complexes to catalyze adenylate m6A modification on RNA.^[^ ^17^ ^]^ METTL3 plays a catalytic role in transferring the methyl group of S‐adenosyl methionine (SAM), while METTL14 provides structural support.^[^ ^18^ ^]^ Additionally, METTL16 is responsible for m6A formation in U6 snRNA^[^ ^19^ ^]^ and also participates in maintaining homeostasis of SAM in an m6A‐dependent manner.^[^ ^20^ ^]^ Methylation readers, such as YTHDF1//2/3, YTHDC1/2, IGF2BP1/2/3, HNRNPC, and RBMX (also known as HNRNPG), are RNA‐binding proteins that specifically bind to the region of m6A modification. They have the ability to alter the secondary structure of RNA and impact the interaction between RNA and protein.^[^ ^7^ ^]^ Different species utilize different m6A reader proteins to achieve specific biological functions.^[^ ^7^ ^]^ For example, in order to adapt to a hypoxic environment, mammals living at high altitudes select YTHDF1 over YTHDF2 and YTHDF3 to resist hypoxia‐induced cellular apoptosis in a Keap1‐Nrf2‐AKR1C1 axis‐dependent manner. This indicates that readers of m6A modified RNAs can have both redundant and specific functions depending on different cellular contexts.^[^ ^21^ ^]^ FTO and ALKBH5 are the only two components of demethylases responsible for catalyzing the demethylation reaction of m6A‐modified bases.^[^ ^7^ ^]^ This modification affects all aspects of RNA lifecycle including transcription, subcellular localization, pre‐mRNA splicing, RNA export, mRNA translation, and RNA degradation without altering the base sequence.^[^ ^22^, ^23^, ^24^, ^25^ ^]^ The methylation process begins in the nucleus; however, its functional regulation occurs in cytosol.^[^ ^26^ ^]^ m6A methylation on RNA is a dynamic process with addition of a methyl group being more homeostatic and frequent. In contrast, RNA demethylation is mainly stimulus dependent and occurs under special conditions.^[^ ^15^ ^]^

## 3 m6A Modification and Pulmonary Fibrosis

### 3.1 m6A Modification and Idiopathic Pulmonary Fibrosis

Idiopathic pulmonary fibrosis (IPF) is a common and fatal lung disease characterized by abnormal accumulation of myofibroblasts and excessive deposition of ECM in the lung tissue, leading to gradual impairment of lung function.^[^ ^27^ ^]^ The majority of IPF patients experience rapid clinical deterioration with a median survival of only 2–3 years, ultimately succumbing to respiratory failure due to decreased respiratory function and progressive dyspnea.^[^ ^28^ ^]^ The pathogenesis of IPF is not thoroughly understood, and current therapies are limited to reducing the rate of pulmonary functional decline in some patients.^[^ ^29^ ^]^ Although m6A modification has been implicated in various biological processes, its role in pulmonary fibrosis remains unclear.

Several studies have demonstrated the significant role of m6A modification in the occurrence and progression of IPF. A study on the risk of genome‐wide genetic correlation between IPF and severe COVID‐19 revealed that genes related to severe acute respiratory syndrome of COVID‐19 infection are broadly regulated by m6A RNA modification in bronchoalveolar lavage cells of 176 IPF patients from the GEO database.^[^ ^30^ ^]^ Global m6A modification is upregulated in lung tissue samples from IPF patients, a mouse model of bleomycin (BLM)‐induced pulmonary fibrosis. Lowering m6A levels through silencing METTL3 inhibits the FMT process via m6A modification on KCNH6 mRNA.^[^ ^31^ ^]^ KCNH6 encodes a member of voltage‐dependent potassium (Kv) channels, which play a pivotal role in regulating the resting membrane potential. Additionally, they are responsible for governing cell proliferation and differentiation, including the m6A‐regulated FMT process.^[^ ^32^ ^]^ Exposure to PM2.5 led to the upregulation of METTL3 expression,^[^ ^33^, ^34^ ^]^ thus promoting m6A modification on the mRNA of CDH1 and Nrf2. The mRNA of CDH1 is bound by YTHDF2, triggering EMT progression in pulmonary fibrosis,^[^ ^33^ ^]^ while the mRNA of Nrf2 is recognized by YTHDF1/IGF2BP1, producing a protective function against oxidative stress in pulmonary fibrosis.^[^ ^34^ ^]^ In a murine model of arsenite‐induced IPF, an increase in m6A modification on the mRNA of neuronal protein 3.1 (NREP) was found to be regulated by H3K18la/YTHDF1. This indicates that m6A‐modified NREP plays a crucial role in the FMT process associated with arsenite‐induced IPF.^[^ ^35^ ^]^ The accumulation of DNA damage in the lung induces cellular senescence and promotes age‐related diseases such as IPF. YTHDC1 activates ataxia telangiectasia and RAD3‐related (ATR) kinase, facilitating DNA damage repair. This indicates a noncanonical function of YTHDC1 in delaying cellular senescence related to pulmonary fibrosis, thus highlighting its potential role in the progression of age‐related lung diseases.^[^ ^36^ ^]^

### 3.2 m6A Modification and Chronic Obstructive Pulmonary Disease

Chronic obstructive pulmonary disease (COPD) is a comprehensive term encompassing various pathological changes in the lungs resulting from gene–environment interactions. Patients with COPD exhibit pathological alterations, such as the loss of small pulmonary vessels, increased permeability of pulmonary microvasculature, and development of emphysema. These changes ultimately lead to a gradual and irreversible restriction of airflow.^[^ ^37^, ^38^ ^]^

Preformation of performed MeRIP‐Seq revealed that the m6A modification of 430 genes increased and 3995 genes decreased in the lung tissues during the stable phase of COPD. Additionally, 740 genes increased and 1373 genes decreased in the lung tissues during the acute exacerbation phase of COPD induced by LPS infusion in mice.^[^ ^39^ ^]^ The results of the KEGG analysis demonstrated a significant enrichment of differentially methylated mRNAs in the interleukin‐17 (IL‐17) signaling pathway, tumor necrosis factor (TNF) signaling pathway, and nuclear factor kappa B (NF‐kB) signaling pathway. This indicates that m6A methylation plays a crucial immunomodulatory role in COPD.^[^ ^39^ ^]^

METTL16‐mediated m6A modification on mRNA of sulfatase‐2 (Sulf2) and its translation plays a critical role in the development of PM2.5‐induced pulmonary microvascular injury and is involved in the pathogenesis of COPD in rats.^[^ ^37^ ^]^ Bioinformatics analysis using Series Matrix Files of gene array expression in small‐airway epithelium from human cigarette smokers has revealed a significant association between m6A RNA methylation regulators (such as IGF2BP3, FTO, METTL3, and YTHDC2) and the development of COPD. This indicates that m6A RNA methylation plays a contributory role in the occurrence of COPD.^[^ ^40^ ^]^ The expression of METTL3 was significantly increased in the lung tissue of patients with cigarette smoking‐induced COPD. Silencing METTL3 improved the EMT process in human bronchial epithelial cells (HBECs) caused by cigarette smoke extract (CSE) through m6A modification of SOCS3 mRNA.^[^ ^41^ ^]^ The m6A modification on circSAV1 (has_circ_0007101) triggers ferroptosis in COPD by recruiting YTHDF1 to promote the translation of iron‐responsive element‐binding protein 2 (IREB2) through the formation of a ternary complex of circSAV1/YTHDF1/IREB2. The elevation of IREB2 contributes to the accumulation of a labile iron pool and lipid peroxidation, ultimately promoting airway remodeling and emphysema.^[^ ^42^ ^]^ ZC3H13, a member of the m6A writers, has been found to enhance the expression and mRNA stability of integrin α6 (ITGA6) through m6A modification. This enhancement has been shown to exacerbate symptoms of COPD, including inflammation, apoptosis, and EMT in human normal lung epithelial cells as well as in a mouse model of COPD induced by tobacco smoke.^[^ ^43^ ^]^

### 3.3 m6A Modification and Silicosis

Silicosis is a significant occupational hazard worldwide, characterized by inflammation of lung tissue and irreversible pulmonary fibrosis caused by crystalline silicon dioxide (SiO2).^[^ ^44^ ^]^ Despite the fact that the underlying mechanisms and pathogenesis remain unknown, m6A methylation has recently garnered attention.

The levels of global m6A methylation and METTL3 expression were found to be upregulated in the lung tissues of mouse silicosis models, while the expression levels of ALKBH5, FTO, YTHDF1, and YTHDF3 were downregulated.^[^ ^45^, ^46^ ^]^ GO analysis revealed that the m6A methylation genes were primarily associated with “protein binding” and “ion binding.” Additionally, KEGG pathway analysis demonstrated that m6A modification may play a role in various biological processes including metabolism, immunity, and cell death in silicosis.^[^ ^46^ ^]^ Stimulation of SiO_2_ induces m6A modification of hsa_circ_0000672 and hsa_circ_0005654 via METTL3 in pulmonary fibroblasts. hsa_circ_0000672 and hsa_circ_0005654 collaboratively target eIF4A3 to enhance the proliferation, migration, and activation of pulmonary fibroblasts, thereby contributing to the development of pulmonary fibrosis.^[^ ^47^ ^]^ Exposure to silica leads to an increase in global m6A modifications in mouse lung tissues through the downregulation of ALKBH5. This downregulation modulates Slamf7 m6A modification and subsequently affects the function of macrophage autophagy via the PI3K/AKT signaling pathway.^[^ ^48^ ^]^ ALKBH5 controls circZNF609 expression and cytoplasmic export in an m6A‐dependent manner to downregulate circZNF609 in silica‐induced fibrotic pulmonary tissues in mice. Overexpression of circZNF609 inhibited fibroblast activation and attenuated lung fibrosis through the miR‐145‐5p/KLF4 axis as well as circZNF609‐encoded peptides.^[^ ^49^ ^]^ ALKBH5 also promoted silica‐induced pulmonary fibrosis via the miR‐320a‐3p/FOXM1 axis or by targeting FOXM1 directly in an m6A‐dependent manner.^[^ ^50^ ^]^ Single‐cell sequencing analysis also revealed that FTO mRNA expression was downregulated in epithelial cells, endothelial cells, fibroblasts, and monocytes after exposure to silica.^[^ ^45^ ^]^

### 3.4 m6A Modification and Asthma

Asthma is a chronic inflammatory immune‐related airway disorder characterized by airway inflammation and remodeling.^[^ ^51^ ^]^ Airway remodeling involves the proliferation or hypertrophy of airway smooth muscle cells, thickening of the subepithelial reticular lamina, matrix deposition throughout the airway wall, angiogenesis, and epithelial mucous metaplasia. These changes lead to airway hyperresponsiveness and a history of respiratory symptoms such as wheezing, shortness of breath, chest tightness, and cough.^[^ ^52^, ^53^ ^]^

A recent study investigated the m6A methylomic landscape in the lung tissues of ovalbumin‐induced acute asthma mice and identified 127 hypermethylated and 43 hypomethylated differentially expressed mRNAs, highlighting the essential role of m6A methylation in the pathogenesis of asthma.^[^ ^54^ ^]^ The expression of miR‐143‐3p was found to be decreased in both asthma patients and TGF‐β1‐treated human bronchial epithelial 16HBE cells, as well as human lung fibroblast HPF cells. Interference of METTL3 reduced the expression of miR‐143‐3p, inhibiting airway epithelial cell EMT and lung fibroblast ECM production via the METTL3/miR‐143‐3p/Smad3 axis.^[^ ^55^ ^]^ Low‐expression METTL3 was observed in monocyte‐derived macrophages from childhood allergic asthma patients. Loss of METTL3 impaired the m6A‐YTHDF3‐dependent degradation of PTX3 mRNA, while higher PTX3 expression positively correlated with asthma severity through promoting M2 macrophage activation.^[^ ^56^ ^]^ These results illustrate that METTL3/YTHDF3‐m6A/PTX3 interactions contribute to macrophage homeostasis and may provide therapeutic targets in the treatment of allergic asthma.^[^ ^56^ ^]^ A lower level of METTL3 was also observed in the peripheral blood of T2 asthma patients and in BALF cells of T2 asthma mice. It was found that METTL3 attenuated Th2 cell differentiation in T2 asthma by modulating the m6A methylation activity of SOX5 in bronchial epithelial cells.^[^ ^57^ ^]^ Additionally, overexpression of METTL3 significantly increased the m6A levels of human β defensin‐3 (HBD‐3) and reduced the mRNA expression and stability of HBD‐3 in PDGF‐BB‐treated ASMCs. This suggests that METTL3‐mediated HBD‐3 is involved in the progression of asthma.^[^ ^58^ ^]^ Exosomal lncRNA PAET has been found to promote the accumulation and stability of METTL3 in PM2.5‐treated HBE cells from children with asthma. This leads to an increase in m6A modification of cytochrome c oxidase subunit 4I1 (COX4I1), while levels of COX4I1 are reduced through a mechanism dependent on YTHDF3, ultimately resulting in DNA damage due to disruption of oxidative phosphorylation. The mechanistic pathway involving exosomal lncRNA PAET‐METTL3‐ YTHDF3‐COX4I1 axis contributes to elevated levels of DNA damage, providing new insights and potential strategies for childhood asthma.^[^ ^59^ ^]^

In primary human airway epithelium, FTO regulates motile ciliogenesis and stabilizes the mRNA encoding the master ciliary transcription factor FOXJ1. In FTO gene knockout mice, strong asthma‐like phenotypes were observed upon allergen challenge due to defective ciliated cells in the airway epithelium.^[^ ^60^ ^]^ Downregulation of FTO expression ameliorated pathophysiological alterations, including attenuated damage to the airway epithelial barrier.^[^ ^61^ ^]^ Activation of FTO in response to oxidative stress impairs the epithelial barrier by upregulating IKBKB, thereby contributing to airway remodeling through m6A‐dependent mRNA stability in a mouse model of asthma.^[^ ^62^ ^]^

YTHDF1 is prominently expressed in airway epithelial cells of allergic individuals and asthmatic patients. In a manner dependent on m6A, YTHDF1 enhances CLOCK translation and triggers the production of NLRP3 inflammasome and secretion of interleukin‐1β, leading to inflammatory responses in the airways.^[^ ^63^ ^]^

## 4 m6A Modification and Cardiac Fibrosis

Cardiac fibrosis is a common pathological change in multiple heart diseases, characterized by abnormal proliferation of cardiac fibroblasts (CF) and deposition of ECM. Myocardial infarction is a typical example of reparative fibrosis, as the sudden death of a large number of cardiomyocytes stimulates inflammation and subsequent activation of reparative myofibroblasts, leading to scar formation.^[^ ^64^ ^]^ In other cardiac diseases such as systemic hypertension, aging, obesity, and diabetes, fibrosis mainly involves the interstitium and develops gradually without significant loss of cardiomyocytes. These cardiac diseases are associated with progressive interstitial and perivascular deposition of ECM proteins that increase myocardial stiffness, leading to cardiac dysfunction and cardiac failure.^[^ ^64^, ^65^ ^]^

Increased m6A methylation has been observed in human and mouse cardiomyopathy.^[^ ^66^, ^67^ ^]^ Alteration of METTL3 expression impacts cell size and cellular remodeling in stressed cardiomyocytes, regulating translational efficiency by affecting transcript stability.^[^ ^67^, ^68^ ^]^ Additionally, METTL3 and lncRNA MetBil were significantly increased in fibrotic tissue following myocardial infarction and interact to dysregulate genes associated with fibrosis pathways.^[^ ^69^ ^]^ The expression level of METTL3 was found to be upregulated in the cardiac fibrotic tissue of mice with chronic myocardial infarction, promoting CF proliferation, transition, and collagens accumulation.^[^ ^70^ ^]^ Silencing METTL3 induced downregulation of IGFBP3 expression and inhibited human primary CFs treated with TGF‐β1.^[^ ^71^ ^]^

METTL3 exacerbates cardiac myocyte proliferation, infarct size, and cardiac function after ischemic myocardial damage by binding to miR‐143‐3p^[^ ^72^, ^73^ ^]^ and miR‐17‐3p^[^ ^74^ ^]^ through m6A modification. Deletion of abraxas brother 1 (ABRO1), a component of the deubiquitinating system, enhances cardiac regeneration and improves heart function after myocardial infarction by METTL3‐mediated m6A methylation of PSPH mRNA and downstream CDK2.^[^ ^75^ ^]^ Knockdown of METTL3 suppresses glycolysis and inhibits cardiac fibrosis by repressing androgen receptor expression in a YTHDF2‐dependent manner.^[^ ^76^ ^]^ Recent studies have shown that m6A modification is involved in mitochondrial fission, with METTL3 potentially regulating the stability and translation of mitochondrial encoded transcripts.^[^ ^77^ ^]^ METTL3 promotes mitochondrial fission by enhancing the methylation of lncRNA GAS5 in a YTHDF2‐dependent manner, leading to the development of myocardial fibrosis.^[^ ^78^ ^]^ Inhibiting METTL3 in a mouse model of myocardial ischemia‐reperfusion injury (MIRI) alleviates cardiac fibrosis inflammation and prevents cardiomyocyte death during reperfusion injury by disrupting DNA‐PKcs/Fis1‐dependent mitochondrial fission, resulting in improved cardiac function.^[^ ^79^ ^]^ METTL3 inhibits autophagic flux and promotes apoptosis in cardiomyocytes via methylation of TFEB, indicating the crucial role of m6A modification in cardiac ischemia.^[^ ^80^ ^]^ In addition, METTL3 deficiency boosts cardiomyocyte proliferation and accelerates heart regeneration in postnatal mice. Conversely, overexpression of METTL3 hinders cardiomyocyte proliferation by downregulating fibroblast growth factor‐16 (Fgf16) expression in an m6A‐YTHDF2‐dependent manner.^[^ ^81^ ^]^ Furthermore, METTL3 plays a role in cardiac fibrosis and cardiomyocyte apoptosis by increasing m6A levels of Tenascin‐C (TNC), an ECM glycoprotein, promoting TNC mRNA stability.^[^ ^82^ ^]^

Knockdown of WTAP improved oxidative stress and preserved cardiac function in ischemic myocardial tissues by inhibiting m6A modification of TXNIP mRNA.^[^ ^65^ ^]^ WTAP is essential for heart development and cardiac function by maintaining the chromatin accessibility of cardiac genes.^[^ ^83^ ^]^

FTO‐dependent cardiac m6A RNA methylation plays a crucial role in regulating cardiac contraction during heart failure. Berulava et al. found that ≈25% of transcripts in healthy mouse and human hearts have m6A RNA methylation. Changes in m6A RNA methylation surpass changes in gene expression during the progression to heart failure in both mice and humans, with altered m6A RNA methylation mainly associated with metabolic and regulatory pathways. Modulating the m6A RNA system through cardiomyocyte‐specific knockout of the demethylase FTO resulted in faster progression of heart failure, leading to reduced ejection fraction and increased dilatation.^[^ ^84^ ^]^ Mathiyalagan et al. recently investigated the functional effect of FTO on cardiac contractile function using clinical human samples, preclinical pig and mouse models, as well as primary cardiomyocyte cell cultures. Reduced FTO expression is observed in heart failure, resulting in an abnormal increase in global cardiac m6A as well as specifically within contractile transcripts. Overexpression of FTO prevented degradation of cardiac contractile transcripts under ischemic conditions by selective demethylation, thereby protecting cardiomyocyte contractile function and mitigating maladaptive cardiac remodeling following MI. Increased FTO expression also alleviates hypoxia‐induced cardiomyocyte dysfunction and restores calcium handling and sarcomere dynamics.^[^ ^85^ ^]^

ALKBH5 levels increase after heart ischemia and help maintain angiogenesis in endothelial cells. It plays a crucial role in this process by reducing m6A modification of sphingosine kinase‐1 (SPHK1), a key intracellular and extracellular messenger that regulates various aspects of vascular biology and physiology.^[^ ^86^ ^]^ Zhang et al. found that decreased ALKBH5 expression is associated with reduced protective effects of hypoxic postconditioning in senescent cardiomyocytes.^[^ ^87^ ^]^ However, ALKBH5 promotes FMT by removing the m6A modification of ErbB4, protecting against cardiac rupture during hypoxia in mice.^[^ ^88^ ^]^ Heart failure downregulated FTO levels in heart tissue after physical training could negate the health benefits of exercise by promoting myocardial fibrosis, myocyte apoptosis, and hypertrophy when overexpressed.^[^ ^89^ ^]^

The m6A modification of total RNA was significantly increased in mice with myocardium hypertrophy, and the expressions of FTO and WTAP were downregulated.^[^ ^90^ ^]^ FTO overexpression inhibited apoptosis of hypoxia/reoxygenation‐treated myocardial cells by regulating m6A modification of Mhrt,^[^ ^89^ ^]^ a cardiac‐specific lncRNA transcribed from the antisense strand of myosin heavy chain gene (MYH7).^[^ ^91^ ^]^ Additionally, circCELF1 (hsa_circ_0095920) has been shown to promote FTO expression and inhibit myocardial fibrosis by regulating the expression of DKK2, an antagonist of Wnt/β‐catenin signaling pathway that inhibits various fibrotic processes through the FTO/DDK2 pathway.^[^ ^92^ ^]^ These findings suggest that FTO may be a potential target for HF treatment.

FTO also plays a role in the heart toxicity caused by doxorubicin, a common chemotherapy for cancer. The FTO protein was significantly reduced in the hearts of mice treated with doxorubicin. FTO helped alleviate doxorubicin‐induced heart toxicity and prevented cell death through ferroptosis by activating P21/Nrf2 via mediating the m6A demethylation of P53 or P21/Nrf2 in a HuR‐dependent manner.^[^ ^93^ ^]^ In addition, circ‐ZNF609 was upregulated through RNA m6A methylation in response to doxorubicin‐induced cardiotoxicity. FTO functioned as the downstream factor of circ‐ZNF609 following doxorubicin stimulation, and inhibition of FTO degradation by circ‐ZNF609 knockdown protected the heart against doxorubicin‐induced cardiotoxicity.^[^ ^94^ ^]^ Furthermore, inhibition of METTL3 alleviated iron accumulation and ferroptosis in cardiomyocytes induced by doxorubicin, while overexpression of METTL3 had the opposite effects. METTL3 promoted m6A modification of TFRC mRNA and enhanced its stability through recognition by IGF2BP2. Pharmacological administration of STM2457, a specific METTL3 inhibitor, effectively ameliorated DIC in mice.^[^ ^95^ ^]^

Endotoxemia can cause a dangerous immune and cardiovascular reaction, leading to tissue damage. In mice, LPS‐induced endotoxemia decreased FTO expression and increased m6A modification on RNAs. Knocking down FTO in cardiomyocytes had similar effects, causing a significant increase in the expression of myocardial inflammatory cytokine genes such as IL‐6, TNF‐α, and IL‐1β. These findings suggest that FTO‐regulated m6A modification plays a critical role in the expression of cardiac proinflammatory cytokines, myocardial inflammation, and cardiac dysfunction during endotoxemia.^[^ ^96^ ^]^

YTHDF2 is involved in various biological processes, including migration, invasion, metastasis, proliferation, apoptosis, cell cycle, viability, adhesion, differentiation, and inflammation in numerous diseases.^[^ ^97^ ^]^ Studies have shown that YTHDF2 levels are significantly increased during the development of heart failure in human samples and a mouse model induced by TAC surgery.^[^ ^98^ ^]^ Overexpression of YTHDF2 reduces cardiac fibrosis and hypertrophy, while knockdown of YTHDF2 has the opposite effect. Further experiments revealed that YTHDF2 suppresses cardiac hypertrophy by inhibiting the expression of Myh7 (myosin heavy chain 7) and Eef2 (eukaryotic elongation factor 2) mRNA through an m6A‐dependent mechanism.^[^ ^99^ ^]^ Loss of YTHDF2 promotes cardiac hypertrophy and dysfunction in a mouse model by binding to m6A‐modified mRNA of myocardial zonula adherens protein (MYZAP).^[^ ^100^, ^101^ ^]^ Additionally, lncRNA MIAT regulates cardiac hypertrophy through its interaction with YTHDF2/PPARα/CPT‐1a axis.^[^ ^102^, ^103^ ^]^ YTHDC1 knockout in mice caused dilated cardiomyopathy, characterized by enlarged left ventricle and disorganized sarcomere arrangement, leading to decreased myocardial contractility and severe systolic dysfunction.^[^ ^104^ ^]^ YTHDC1 binds to m6A‐modified Titin mRNA and its loss enhances the expression ratio of N2BA to N2B isoforms of Titin. YTHDC1 regulates contractile function and the development of dilated cardiomyopathy by facilitating the inclusion of exons that code for the more compliant N2BA isoform.^[^ ^104^ ^]^ IGF2BP2 expression remains low in adulthood, but increases in patients with dilated cardiomyopathy or myocardial infarction.^[^ ^105^ ^]^ Studies in mice have shown that upregulation of IGF2BP2 leads to cardiac remodeling, fibrosis, heart failure, and eventual death, while downregulation of IGF2BP2 has potential in rescuing heart injury.^[^ ^105^ ^]^ Additionally, it was demonstrated that IGF2BP2 collaborates with miR‐133a and AGO2 in an RNA methylation‐dependent manner, leading to translational repression and contributing to cardiac hypertrophy.^[^ ^106^ ^]^ hnRNPC is up‐regulated and relocated to the sarcomeric Z‐disc during ECM remodeling in human myocardium from heart failure patients and in a mouse model of myocardial infarction.^[^ ^107^ ^]^ Interactions between hnRNPC and its mRNA targets are important for maintaining cardiomyocyte integrity and cardiac contractility.^[^ ^108^ ^]^

Diabetes is associated with cardiomyopathy and an increased risk of heart failure. A prominent feature of diabetic cardiomyopathy is the presence of cardiac interstitial and perivascular fibrosis, which may contribute to the development of cardiac dysfunction.^[^ ^109^, ^110^ ^]^ Both m6A modification and FTO expression were reduced in a mouse model of diabetes. Overexpression of FTO improved cardiac function by attenuation myocardial fibrosis and myocyte hypertrophy through the demethylation of m6A on a series of mRNAs.^[^ ^111^ ^]^ Additionally, it was observed that a high‐fat diet led to reduced FTO expression and increased METTL3 expression in the heart tissues of rats with cardiomyopathy. However, intermittent fasting was able to reverse these effects, suggesting that m6A RNA methylation plays critical roles in obesity‐related cardiopathy.^[^ ^112^ ^]^ In a putative model of cardiac fibrosis associated with type 2 diabetes, the upregulation of WTAP was found to promote CF proliferation and migration, contributing to diabetic cardiac fibrosis. Conversely, knocking down WTAP suppressed mitochondrial lipid oxidation, as well as fibroblast proliferation and migration, ultimately attenuating diabetic cardiac fibrosis. Clinical observations also revealed that increased levels of WTAP and YTHDF2 were correlated with decreased expression of the androgen receptor in human‐dilated cardiomyopathy heart tissue.^[^ ^113^ ^]^ Moreover, it has been observed that cardiac ischemic tolerance can be enhanced through dietary interventions such as fasting, which leads to the upregulation of FTO and ALKBH5. This is associated with significant alterations in myocardial gene expression.^[^ ^114^ ^]^

## 5 m6A Modification and Hepatic Fibrosis

Hepatic fibrosis is a pathological process that occurs in various chronic liver conditions, characterized by long‐term liver injury‐activating hepatic stellate cells (HSCs). These cells undergo morphological and functional changes, transforming into fibroblasts and secreting large amounts of ECM, leading to excessive deposition in the interstitium.^[^ ^115^ ^]^ Hepatic fibrosis results from chronic damage repair, including viral hepatitis, nonalcoholic steatohepatitis, parasitemia, inborn errors of metabolism, and toxic damage from alcohol consumption. If left untreated, it can progress to cirrhosis or even hepatic cancer and may result in death.^[^ ^116^ ^]^

Two studies were conducted to investigate the m6A profiles in CCL4‐induced liver fibrosis. The first study identified 995 highly conserved differential m6A peaks associated with 2025 genes involved in regulating diverse pathways,^[^ ^117^ ^]^ while the second study identified 3315 genes with differential m6A levels closely linked to liver fibrosis‐related pathways such as endoplasmic reticulum stress, PPAR signaling pathway, and TGF‐β signaling pathway.^[^ ^118^ ^]^ These findings suggest that m6A methylation is closely linked to the occurrence and development of liver fibrosis and may impact its progression by regulating critical transcripts. Additionally, a study on Wilson's disease found 1913 genes with significantly different levels of m6A methylation, indicating a potential association between m6A modification and the development of liver fibrosis caused by Wilson's disease.^[^ ^119^ ^]^

A genome‐wide expression study was conducted to compare the expression of m6A regulators in liver biopsies from NAFLD patients and healthy controls. The results showed that NAFLD liver samples exhibited increased expression of METTL3, METTL14, FTO, and EIF3H, while the expression of WTAP, RBM15, YTHDC1, YTHDC2, IGF2BP2, HNRNPC, and HNRNPA2B1 was decreased. Furthermore, it was found that RBM15, YTHDC2, HNRNPC, HNRNPA2B1, and EIF3H were correlated with steatosis; KIAA1429 and YTHDF1 were correlated with the degree of lobular inflammation. These findings indicate that dysregulation of m6A methylation contributes to steatosis and fibrosis development in NAFLD.^[^ ^120^ ^]^

Upregulation of METTL3/METTL14 and global m6A hypermethylation were observed in LPS‐activated Kupffer cells (KCs) and in the liver of rats with HFD (high‐fat diet)‐induced NASH. LPS‐responsive m6A peaks were identified on TGF‐β1 mRNA, and NF‐κB directly transactivates METTL3 and METTL14 genes. Additionally, LPS‐stimulated TGF‐β1 expression is abolished in METTL3/METTL14‐deficient KCs and in METTL14 knockout mice. The increased m6A modification on TGF‐β1 mRNA promotes progression from NASH to fibrosis in KCs and the liver of HFD‐induced NASH rats.^[^ ^121^ ^]^

ASIC1a (acid‐sensitive ion channel 1a) regulates the processing of miR‐350 through METTL3‐dependent m6A modification, and mature miR‐350 targets SPRY2 to promote liver fibrosis via the PI3K/KT and ERK pathways.^[^ ^122^ ^]^ Silencing of METTL3 resulted in a reduction of m6A deposition on mRNA transcripts of Lats2, a central player in the Hippo/YAP signaling pathway, inhibiting their degradation. Elevated Lats2 promoted phosphorylation of YAP transcription factor YAP, suppressed YAP nuclear translocation, and reduced pro‐fibrotic gene expression. Disruption of METTL3 ameliorated liver fibrosis by controlling the Hippo/YAP signaling pathway.^[^ ^123^ ^]^ Proinflammatory M1 macrophages, through the activation of HSCs, play a role in contributing to liver fibrosis. It was found that the METTL3/MALAT1/PTBP1/USP8/TAK1 axis promoted pyroptosis and inflammation in macrophages, exacerbating liver fibrosis. Targeting individual components within this axis may be beneficial for treatment.^[^ ^124^ ^]^ Overexpression of METTL3 suppressed miR‐146a‐5p maturation and induced apoptosis, increased proinflammatory cytokines, HBsAg and HBeAg levels, and inhibited cell proliferation in THLE‐2 cells. Knockdown of METTL3 hindered miR‐146a‐5p maturation in HBV‐infected mice, improving liver injury in mice with HBV‐associated hepatic failure.^[^ ^125^ ^]^

The expression of METTL14 and m6A modification were reduced in hepatic tissue from patients with liver fibrosis. Knockdown of METTL14 promoted HSCs activation and worsened liver fibrosis in mice. METTL14 exerts m6A modification on NOVA2 (ventral antigen 2) mRNA by binding to its 3'UTR region in the nucleus, leading to degradation by YTHDF2 in the cytoplasm. The METTL14‐YTHDF2‐NOVA2 axis plays a crucial role in regulating LF progression.^[^ ^126^ ^]^ METTL14 levels are significantly reduced in MAFLD patients and mouse models, exacerbating lipid accumulation, fibrosis, and liver injury through decreased GLS2 levels. Restoring METTL14 could alleviate liver injury and fibrosis through the CX3CR1/MyD88/NF‐κB/S100A4 signaling pathway.^[^ ^127^ ^]^

The expression of METTL3 decreases with liver impairment in a time‐dependent manner, suggesting its involvement in CdCl2‐induced hepatotoxicity. Cadmium is a widespread environmental pollutant known for its adverse effects on the liver. Overexpression of METTL3 in hepatocytes mitigates CdCl2‐induced steatosis and liver fibrosis, shedding light on its role in responding to environmental stimuli.^[^ ^128^ ^]^

DHA, a natural and safe antimalarial drug with multiple pharmacological activities, attenuates liver fibrosis by inducing ferroptosis in HSCs. It downregulates the demethylase FTO and increases m6A modifications in BECN1 mRNA via YTHDF1, leading to activation of autophagy and ultimately promoting ferroptosis of HSCs.^[^ ^129^, ^130^ ^]^ ALKBH5 is significantly downregulated in fibrotic liver tissues from both humans and mice, and its overexpression reduces proliferation and migration of HSCs induced by CCl4 in mouse models of liver fibrosis.^[^ ^131^ ^]^ Radiation‐induced activation of ALKBH5 mediates the demethylation of TIRAP mRNA and enhances its expression, thus promoting the occurrence of radiation‐induced liver fibrosis (RILF) through JNK/Smad2 and NF‐κB pathways.^[^ ^132^ ^]^ In a mouse model of CCl4‐induced liver fibrosis, global m6A levels were increased while ALKBH5 expression declined significantly. Furthermore, ALKBH5 mediates the m6A demethylation dynamin‐related protein 1 (Drp1) mRNA in an YTHDF1‐independent manner, suggesting a potential demethylation‐based approach for liver fibrosis diagnosis and therapy.

AcSDKP reduces WTAP expression and inhibits apoptosis through the Hedgehog pathway in CCl4‐induced rat HSCs. Specifically, WTAP targets Ptch1 mRNA's 3'‐UTR, and AcSDKP administration decreases Ptch1 mRNA stability in an m6A‐dependent manner. These findings suggest that the AcSDKP/WTAP/m6A/Ptch1 axis plays a crucial role in rat liver fibrosis.^[^ ^133^ ^]^

The liver clock gene NR1D1 (nuclear receptor subfamily 1 group d member 1) is dysregulated in CCl4‐induced liver fibrosis in mice. NR1D1‐deficient mice are more sensitive to CCl4‐induced liver fibrosis, indicating its crucial role in the progression of fibrosis. YTHDC1 promotes the degradation of NR1D1 mRNA, inhibiting mitochondrial fission function and increasing mitochondrial DNA release in HSCs.^[^ ^134^ ^]^

YTHDF1 enhances mRNA stability by hypermethylating RNA m6A on the transcripts of major collagen genes, thereby alleviating CCl4‐induced liver fibrosis and massive ECM production in mice through HSC‐specific inhibition of collagen production.^[^ ^135^ ^]^ Overexpression of circIRF2 has been shown to attenuate liver fibrogenesis and activation of HSCs during the fibrogenesis stage, while downregulation of circIRF2 exacerbates liver injury and inflammation in mice. It has been identified that YTHDF2 recognizes m6A‐modified circIRF2 and subsequently reduces its stability, contributing to decreased levels of circIRF2 in liver fibrosis. Additionally, it has been observed that circIRF2 may directly interact with miR‐29b‐1‐5p, competitively relieving its inhibitory effect on FOXO3, inducing FOXO3 nuclear translocation and accumulation.^[^ ^136^ ^]^ YTHDF3 was upregulated in the liver tissue of patients with chronic fibrosis and CCl4‐induced mice. Peroxiredoxin 3 (PRDX3), a peroxidase localized in the mitochondria known to play a key role in regulating mitochondrial oxidative stress and providing hepatoprotection, is regulated by YTHDF3 specifically at the level of translation and expression, thereby impacting its function during liver fibrosis through the mitochondrial ROS/TGF‐β1/Smad2/3 signaling pathway.^[^ ^137^ ^]^

IGF2BP2 was found to be upregulated in liver fibrosis and was shown to activate HSCs in both human and mouse models of fibrotic liver induced by CCl4. Furthermore, inhibition of IGF2BP2 was found to block HSCs activation and the progression of liver fibrogenesis. It was also discovered that IGF2BP2 increased the stability of the m6A‐modified transcript aldolase A (ALDOA) in the glycolytic metabolic pathway, which plays a crucial role in HSC activation. These findings highlight the essential role of IGF2BP2 in liver fibrosis through its regulation of glycolytic metabolism and suggest that targeting IGF2BP2 may hold potential as a strategy for treating liver fibrosis.^[^ ^138^ ^]^

The m6A modification has also been implicated in explaining the mechanism of action of certain traditional Chinese medicines. Isovitexin (IVT), derived from *Hydrocotyle sibthorpioides*, has been shown to reduce collagen deposition and HSC activation in a mouse model of liver fibrosis induced by CCl4 as well as in LX2 and JS‐1 cell lines treated with platelet‐derived growth factor‐BB (PDGF). IVT was found to increase pri‐miR‐21 levels and reduce the m6A enrichment of pri‐miR‐21 via m6A modification, thereby alleviating hepatic fibrosis through the miR‐21‐mediated PI3K/Akt signaling pathway and glutathione metabolic pathway.^[^ ^139^ ^]^

## 6 m6A Modification and Renal Fibrosis

Renal fibrosis is the main pathological process in kidney diseases, characterized by excessive accumulation of myofibroblasts and ECM in the renal interstitium and glomeruli. Both acute kidney injury (AKI) and chronic kidney disease (CKD) are linked to renal fibrosis.^[^ ^140^, ^141^ ^]^ It plays a crucial role in the development of CKD, including obstructive nephropathy (ON), diabetic nephropathy (DN), and hypertensive nephropathy, ultimately leading to high morbidity and mortality rates associated with various renal disease complications.^[^ ^142^ ^]^

The global levels of m6A were found to be increased in the kidneys after bilateral ureteral obstruction in young rats, while the mRNA expression levels of m6A methyltransferases and demethylases were significantly decreased. Additionally, there was a significant upregulation in the expression levels of EGFR and Brcal, whereas the mRNA expression levels of Notch1 were downregulated. These findings suggest that alterations in the m6A epitranscriptome may potentially play a role in the pathophysiological processes of ON in renal failure due to urinary tract obstruction in children and infants.^[^ ^143^ ^]^

The knockdown of METTL3 in MMC cells reduced m6A RNA methylation levels and proinflammatory cytokines IL6 and TNF‐α, leading to inhibited cell proliferation and cycle progression.^[^ ^144^ ^]^ Inhibition of METTL3 in HK‐2 cells also decreased TGF‐β‐induced fibrotic marker expression. The specific inhibitor STM2457 showed potential in reducing kidney fibrosis in vivo. Furthermore, increased METTL3 protein expression was observed in tissues from CKD patients with diabetic or IgA nephropathy.^[^ ^145^ ^]^

The upregulation of METTL3 enhances the m6A modification of Ena/VASP‐like (EVL, a member of the Enabled/vasodilator stimulated phosphoprotein family) mRNA, leading to improved stability and expression in an IGF2BP2‐dependent manner. Highly expressed EVL binding to Smad7 abolishes Smad7‐induced suppression TGF‐β1/Smad3 signal transduction, accelerating renal fibrosis progression. Targeting the overactivated METTL3/EVL m6A axis may be a potential strategy for treating renal fibrosis.^[^ ^146^ ^]^ In response to AKI stimuli such as TNF‐α, cisplatin, and LPS, METTL3 is highly expressed in tubular epithelial cells (TECs). METTL3 promotes renal inflammation and injury by increasing m6A RNA methylation of TAB3 and enhancing the stability of TAB3 mRNA through IGF2BP2‐dependent mechanisms.^[^ ^147^ ^]^

Levels of METTL3 protein and mRNA were increased in HK2‐cell line and the fibrotic kidney of UUO mouse model, playing a significant role in driving obstructive renal fibrosis development via the miR‐21‐5p‐SPRY1/ERK/NF‐kB axis.^[^ ^148^ ^]^ MALAT1 expression was elevated in renal fibrosis tissues of patients with ON. Knocking down MALAT1 suppressed TGF‐β1‐induced EMT, ECM deposition, and cell viability, proliferation, and migration in HK2 cells.^[^ ^149^ ^]^ Additionally, METTL3 was found to methylate MALAT1 mRNA in TGF‐β1‐treated HK2 cells, potentially influencing the MALAT1/miR‐145/FAK pathway in renal fibrosis.^[^ ^149^ ^]^ Inhibition of METTL3 reduced RNA m6A levels and decreased cell apoptosis during H/R treatment. Upregulation of METTL3 was noted in IRI and H/R models primarily promoting apoptosis by modifying forkhead box D1 (FOXD1), a member of the FOX protein family. The mechanism involving METTL3/m6A/Foxd1 primarily serves to promote apoptosis.^[^ ^150^ ^]^ Furthermore, it was discovered that METTL3 is recruited by lncRNA AI662270 to deposit m6A modifications on mRNA of CTGF, leading to exacerbating kidney fibrosis in both UUO‐and streptozotocin (STZ)‐treated mice.^[^ ^151^ ^]^ METTL14 promotes renal ischemic reperfusion injury through the suppression of YAP1 whereas its suppression confers protection against IRI by inhibiting YAP1‐TEAD signaling with Peptide 17.^[^ ^152^ ^]^

FTO expression was increased in a mouse model of UUO and TGFβ1‐treated TECs. In mice with FTO heterozygous mutation and in cells treated with small interfering RNA (siRNA), the induction of EMT by UUO and TGFβ1 was reduced. This reduction was evidenced by decreased fibronectin and N‐cadherin accumulation, as well as increased levels of E‐cadherin. Silencing FTO also improved inflammation, apoptosis, and inhibition of autophagy induced by UUO and TGFβ1, achieved through demethylation of RUNX1 mRNA and enhancement of its stability via the PI3K/AKT pathway.^[^ ^153^ ^]^ Furthermore, FTO overexpression alleviated TNFα‐induced damage to HK‐2 cells by targeting AQP3 in an m6A‐dependent manner. Conversely, silencing FTO led to opposite results, suggesting that FTO negatively regulates AQP3 levels through an m6A‐dependent mechanism which compromises AQP3 stability.^[^ ^154^ ^]^ The level of TWIK‐related acid‐sensitive K+ channel‐2 (TASK‐2, encoded by Kcnk5) was increased in a mouse model and patients with tubulointerstitial renal fibrosis. Activation of TASK‐2 led to reduced intracellular K+ levels, promoting fibrogenesis. Knockout of Kcnk5 or inhibition of TASK‐2 improved G2/M cell‐cycle arrest and attenuated renal fibrosis. FTO deficiency reduced m6A modification of Kcnk5 mRNA after renal fibrosis, alleviating the upregulation of TASK‐2 and mitigating renal fibrosis.^[^ ^155^ ^]^

Alcoholic kidney damage primarily shows as reduced renal tubular function. Ethanol mediates FTO's impact on PPAR‐α m6A mRNA methylation through YTHDF2. This also leads to the degradation of PPAR‐α by facilitating the binding of YTHDF2, ultimately activating NLRP3 inflammasome and releasing inflammatory cytokines, worsening renal inflammation and injury.^[^ ^156^ ^]^

FTO was upregulated and lncRNA GAS5 was downregulated in TGF‐β1‐treated HK‐2 and HKC‐8 cells as well as in mouse kidney tissue following UUO. Overexpression of lncRNA GAS5 or silencing of FTO suppressed the TGF‐β1‐induced increase in EMT‐related proteins (Vimentin, Snail, and N‐cadherin) and inflammatory cytokines (IL‐6, IL‐1β, TNF‐α) levels in HK‐2 cells. FTO inhibited lncRNA GAS5 expression by reducing its m6A modification. Knockdown of FTO also suppressed the EMT process and inflammation response induced by TGF‐β1.^[^ ^157^ ^]^ Additionally, FTO expression was significantly downregulated in patients with sepsis‐associated AKI. In LPS‐induced AKI using the HK‐2 human TEC line, it was observed that FTO suppressed the m6A modification of lncRNA SNHG14 and inhibited autophagy via regulating miR‐373‐p/ATG7.^[^ ^158^ ^]^

ALKBH5 levels decreased and global m6A levels increased in rats with renal fibrosis induced by unilateral ureteral ligation. Overexpression of ALKBH5 led to an increase in E‐cadherin and a decrease in Snail expression.^[^ ^159^ ^]^ Knockdown of ALKBH5 inhibited cell viability, induced cell apoptosis, and reduced inflammation cytokine production in LPS‐treated HK‐2 cells via m6A modification on MALAT1.^[^ ^160^ ^]^ Furthermore, ALKBH5 deficiency upregulated the level of CCL28 by promoting CCL28 mRNA m6A methylation and its stability. Targeting ALKBH5 and this axis could be a potential treatment for AKI.^[^ ^161^ ^]^

The protein level of WTAP increased in a mice model of AKI induced by cecum ligation puncture (CLP) and promoted the expression of lamin B1 gene (LMNB1) through m6A methylation modification in HK2 cell line. The m6A modification mediated by WTAP led to inflammation, ferroptosis, and mitochondrial damage in LPS‐treated HK‐2 cells by regulating LMNB1 expression through activation of NF‐κB and JAK2/STAT3 pathways.^[^ ^162^ ^]^ Septic AKI is characterized by inflammation. In a mouse model of septic AKI induced by CLP surgery, IGF2BP1 was found to upregulate MIF and induce pyroptosis in renal tubular cells. This process is mediated through direct upregulation of E2F1 expression via m6A modification. These findings suggest that IGF2BP1 could be a potent inducer of pyroptosis in septic AKI, and targeting IGF2BP1 may be an alternative strategy for its treatment.^[^ ^163^ ^]^ YTHDF1 is highly expressed in glomerular mesangial cells treated with TGF‐β, as well as in myofibroblasts showing increased expression of the signature protein α‐SMA. Additionally, elevated levels of YTHDF1 were observed in fibrotic mouse kidneys induced by various factors, indicating its causal role in renal fibrosis. Subsequent studies have shown that YTHDF1 promotes fibrosis by upregulating YAP expression.^[^ ^164^ ^]^

Geniposide (GP), an iridoid compound with known anti‐inflammatory, antioxidant, and antiapoptotic effects, has been shown to enhance global m6A methylation and regulate the expression of the PI3K/AKT3/FOXO1 signaling pathway through m6A modification. This contributes to alleviating cell cycle arrest and apoptosis in HK‐2 cells exposed to H_2_O_2_ oxidative stress.^[^ ^165^ ^]^

The levels of METTL3 and m6A content were significantly elevated in the serum of patients with diabetic kidney disease (DKD), as well as in high‐glucose‐stimulated HK‐2 cells and the kidneys of DKD mice.^[^ ^166^ ^]^ Silencing of METTL3 inhibited the progression of DN by regulating the m6A modification of PINK1, a process that depends on YTHDF2.^[^ ^166^ ^]^ Conversely, overexpression of METTL3 alleviated renal injury and fibrosis in DN by increasing the mRNA of nuclear receptor‐binding SET domain protein 2 (NSD2) and promoting m6A modification of NDS2 mRNA, thereby enhancing its stability via YTHDF1.^[^ ^167^ ^]^ METTL3 expression is increased in podocytes of renal biopsies from DN patients. METTL3 overexpression exacerbates inflammation and apoptosis in high‐glucose‐stimulated podocytes, while knockout of METTL3 attenuates these responses. Silencing METTL3 improves albuminuria and histopathological injury in diabetic mice. These findings suggest that METTL3‐mediated m6A modification of TIMP2, in an IGF2BP2‐dependent manner, exerts proinflammatory and proapoptotic effects, representing an important mechanism of podocyte injury in DN.^[^ ^168^ ^]^ Renal biopsy samples from DN patients exhibit low circ‐0000953 expression, correlating with renal function. Podocyte conditional knockin (cKI) or systemic overexpression of circ‐0000953 reduces albuminuria and restores autophagy in diabetic mice kidneys, while knockdown worsens albuminuria and podocyte injury. METTL3 modifies the expression and methylation level of circ‐0000953 through YTHDF2 to regulate podocyte autophagy by binding to Mir665‐3p‐Atg4b, providing a potential biomarker for preventing and treating DN.^[^ ^169^ ^]^

The levels of METTL14 were found to be upregulated in the kidneys of patients with DN and in high‐glucose‐induced human renal glomerular endothelial cells (HRGECs). Overexpression of METTL14 led to increased levels of reactive oxygen species, TNF‐α, interleukin‐6 (IL‐6), and apoptosis, while silencing METTL14 reversed these effects. METTL14 plays a crucial role in high‐glucose‐induced damage to glomerular endothelial cells through m6A modification of α‐klotho.^[^ ^170^ ^]^ It also exerts an influence on HDAC5 (histone deacetylase 5, a member of II HDAC subfamily)‐mediated EMT in kidney TECs by regulating the PI3K/Akt signaling pathway through m6A modifications of PTEN. Additionally, METTL14 was found to be upregulated in renal biopsy samples from patients with focal segmental glomerulosclerosis and DN, as well as cultured human podocytes subjected to adriamycin.^[^ ^171^ ^]^ Knockdown of METTL14 led to an increase in the level of Sirt1 in podocytes and resulted in improved glomerular function, attenuating podocyte injury by promoting autophagy activation and inhibiting apoptosis and inflammation. These findings suggest that METTL14‐dependent RNA m6A modification contributes to podocyte injury through posttranscriptional regulation of Sirt1 mRNA.^[^ ^172^ ^]^

FTO expression is upregulated in high‐glucose‐induced podocytes, aggravating podocyte injury and inflammation. Knockdown of FTO increases serum amyloid A2 (SAA2) mRNA m6A modification and decreases SAA2 mRNA expression. FTO promotes podocyte injury and inflammation by m6A modification of SAA2 mRNA through activating the NF‐κB signaling pathway.^[^ ^173^ ^]^ FTO expression was significantly reduced in the serum samples of DN patients compared with healthy volunteers and is also significantly decreased in DKD patients. The FTO/SOCS1/JAK‐STAT axis promotes DKD pathogenesis through inflammation promotion. Additionally, overexpression of FTO can greatly alleviate kidney inflammation in mice.^[^ ^174^ ^]^ FTO‐mediated m6A modification induces upregulation of lncRNA ENST00000436340, enhancing the binding of PTBP1 to its target gene RAB3B (Ras‐related protein Rab‐3B), promoting degradation of RAB3B mRNA, leading to podocyte injury and DN progression.^[^ ^175^ ^]^ Canagliflflozin (Cana), a sodium‐glucose cotransporter 2 (SGLT2) inhibitor used to treat DN, protects the kidney from fibrosis by inhibiting FTO and increasing the stability of p62/SQSTM1, a classical selective autophagy receptor.^[^ ^176^ ^]^

CircUBXN7 was upregulated in the plasma of DKD patients and correlated with renal injury. In vitro, it increased macrophage activation, EMT, and fibrosis, while in vivo it promoted macrophage infiltration, EMT, fibrosis, and proteinuria. CircUBXN7 also formed an RNA‐protein complex with IGF2BP2 and transcription factor SP1, enhancing the mRNA stability of SP1 and promoting its expression. This led to enhanced macrophage infiltration and renal fibrosis, accelerating the progression of DKD.^[^ ^177^ ^]^ Meanwhile, IGF2BP3 was found to promote CAMK1 mRNA stability through m6A modification and inhibit mitochondrial fission to repress cell apoptosis in HK‐2 cells treated with high glucose as well as a mouse model of DN induced by streptozotocin treatment. These findings provide new directions for developing therapy targets for DN.^[^ ^178^ ^]^

## 7 Therapeutic Applications of m6A Modification in Treating Fibrotic Diseases of Visceral Organs

The studies indicate that m6A modification is critical in the development of visceral organ fibrosis. Targeting m6A modification in specific organs and cells may offer a novel treatment strategy for fibrosis. Additionally, various drugs and therapies can regulate fibrotic diseases by modulating m6A modification either directly or indirectly (**Table** **2**).

**Table 2:** Applications for fibrotic diseases by regulating m6A modification.

Therapies/reagents	Intervention	Effect	References
AAV6	Inhibiting the FMT process by silencing METTL3	Ameliorating pulmonary fibrosis	[31]
FB32	Inhibiting FTO and alleviating allergic inflammation in epithelial cells	Mitigating allergic inflammation of asthma	[179]
pcDNA3.1 vectors	Silencing Zbtb7b to recruit ALKBH5 and inhibiting m6A modification of IL6 mRNA	Ameliorating radiation‐induced pulmonary fibrosis	[180]
siRNA	Silencing METTL3 and enhancing the protein expression of SOCS3	Inhibiting CSE‐induced EMT process of HBECs	[41]
pAAV‐MCS	Activating ATR and facilitating DNA damage repair by upregulating YTHDC1	Mitigating pulmonary fibrosis	[36]
Lentivirus	Promoting Nrf2 translation and ameliorating oxidative stress by upregulating METTL3	Ameliorating pulmonary fibrosis	[34]
Lentivirus	Downregulating ZC3H13 and influencing bronchial epithelial cell inflammation and fibrosis	Ameliorating COPD	[43]
AAV	Inhibiting circSAV1expresion and ameliorating ferroptosis of lung epithelial cells via recruiting YTHDF1	Attenuating COPD	[42]
AAV9	Increasing circZNF609 and inhibiting fibroblast activation by increasing ALKBH5 expression	Ameliorating lung fibrosis of silicosis	[49]
Cinnamaldehyde	Promoting CYP4F40 expression by increasing METTL3	Alleviating steatosis	[187]
Resveratrol	Reducing m6A abundance in mice liver	Mitigating hepatic disturbance induced by HFD	[188]
Betaine	Decreasing lipogenesis and increasing lipolysis by inhibiting FTO expression	Alleviating HFD‐induced hepatic injury	[189]
MA	Inhibiting FTO activity and reducing m6A modification on mRNAs of lipogenic genes in primary hepatocytes	Preventing triglyceride accumulation in primary hepatocytes	[190]
Entacapone	Reducing the expression of FOXO1 by inhibition of FTO	Regulating gluconeogenesis and thermogenesis in in the liver	[191]
AAV9	Upregulating FTO expression, increasing cardiac glucose uptake and ameliorating the mitochondrial structure disorder	Alleviating ischemic cardiac injury	[181]
AAV9	Increasing FTO expression and improving cardiac contractile dysfunction after cardiac infarction	Alleviating cardiac fibrosis	[85]
Adenovirus	Increasing METTL14 expressing, reducing infarct size and apoptosis	Alleviating cardiac ischemia	[182]
AAV9	Increasing YTHDF2 expression and promoting degradation of Myh7 mRNA	Alleviating cardiac hypertrophy	[98]
Lentivirus	Inhibiting METTL3 expression and reducing cardiomyocyte pyroptosis	Alleviating cardiac ischemia	[73]
STM2457	Inhibiting METTL3 activity, reducing monocyte migration and fibrogenesis	Attenuating cardiac inflammation and fibrosis	[183]
IOX1	Inhibiting ALKBH5 activity, reducing cardiac dysfunction and fibrosis	Ameliorating cardiac ischemia	[184]
LNPs	Inhibiting ALKBH5 expression, altering cardiac macrophage	Improving cardiac fibrosis	[185]
SAH	Inhibiting METTL3 activity, reducing mitochondrial fragmentation and myofibrillar transformation	Improving cardiac infarction	[186]
AAV9	Reducing METTL3 expression, reducing renal injury and inflammation	Ameliorating renal injury	[147]
AAV9	Inhibiting METTL3 expression, reducing podocyte loss	Alleviating renal injury	[168]
Cholesterol‐conjugated siRNA	Inhibiting YTHDF1 expression and upregulating YAP	Ameliorating renal fibrosis	[164]
Genistein	Increasing ALKBH5 expression and inhibiting EMT	Ameliorating renal fibrosis	[159]
Cpd‐564	Inhibiting METTL3 activity, reducing TAB3 expression	Improving AKI injury	[147]
TFA	Enhancing the activation of METTL3, reducing pyroptosis and injury in podocytes	Protecting podocytes in DKD	[192]

As the most common fibrotic disease, there are currently no drugs targeting m6A modification in pulmonary fibrosis. METTL3 silencing via the AAV6 system alleviated BLM‐induced pulmonary fibrosis and inhibited the FMT process in mice.^[^ ^31^ ^]^ FB23, a small‐molecule inhibitor of FTO, reduced allergic inflammation in epithelial cells and house dust mite‐induced mice through various cellular processes and EMT‐signaling pathways, indicating that FTO could be a therapeutic target for asthma management.^[^ ^179^ ^]^ ALKBH5 facilitated IL‐6 mRNA demethylation by silencing Zbtb7b using pcDNA3.1 vectors in THP1 cells, which inhibited its nuclear export and suppressed IL‐6 production in the lung, thereby slowing radiation‐induced pulmonary fibrosis development.^[^ ^180^ ^]^ Silencing METTL3 with double‐stranded siRNAs decreased SOCS3 mRNA's m6A methylation, enhancing SOCS3 protein expression and inhibiting CSE‐induced SOCS3/STAT3/SNAI1 signaling along with EMT processes in HBECs, offering new insights into COPD management.^[^ ^41^ ^]^ Exogenous overexpression of YTHDC1 with pAAV‐MCS mitigated pulmonary senescence and fibrosis independent of its m6A‐binding ability in mice.^[^ ^36^ ^]^ Lentivirus‐mediated upregulation of METTL3 protected against pulmonary fibrosis via m6A modification on Nrf2 mRNA expression in mice.^[^ ^34^ ^]^ Downregulating ZC3H13 with interfering lentivirus effectively reduced lung tissue injury from CSE‐induced COPD.^[^ ^43^ ^]^ Reducing circSAV1 expression using AAV‐circSAV1 shRNA safeguarded against emphysema and airway remodeling by inhibiting ferroptosis through YTHDF1 recruitment to promote IREB2 translation in experimental COPD.^[^ ^42^ ^]^ ALKBH5 regulates circZNF609 expression and cytoplasmic export in an m6A‐dependent manner. Overexpressing circZNF609 via the AAV9 system reduced silica‐induced lung fibrosis in mice.^[^ ^49^ ^]^

In myocardial fibrosis, early therapeutic research targeting m6A modification mainly focused on the effector cells–CFs. Overexpressing FTO via the AAV9 system alleviated cardiac dysfunction and left ventricular hypertrophy in mice after transverse aortic constriction.^[^ ^181^ ^]^ Similarly, sustained FTO expression using AAV9 enhanced cardiac function postmyocardial infarction in mice.^[^ ^85^ ^]^ Tail vein injection of adenovirus carrying METTL14 overexpression plasmids markedly improved cardiac dysfunction during I/R in mice.^[^ ^182^ ^]^ Administration of AAV9 particles with YTHDF2 plasmids through tail vein injection for 3 weeks reduced cardiac hypertrophy, indicated by smaller heart size and less fibrosis after transverse aortic constriction.^[^ ^98^ ^]^ In addition to these therapies, tail vein injection of lentivirus‐packaged sh‐METTL3 vector into I/R rats 24 h before surgery substantially mitigated myocardial injury.^[^ ^73^ ^]^ Targeting m6A modifications on monocytes using METTL3 inhibitor STM2457 delivered by drug‐loaded nanoparticles on erythrocyte microvesicles decreased cardiac inflammation and fibrosis. This sheds light on m6A's role in monocyte‐mediated cardiac fibrosis and opens new avenues for biohybrid treatments related to ventricular septal defect occluder complications and heart diseases.^[^ ^183^ ^]^ Daily caudal vein injections of nanocages loaded with ALKBH5 inhibitor IOX1 for 14 days effectively reduced cardiac dysfunction and fibrillation in acute myocardial infarction models, offering a potential future strategy for AMI treatment.^[^ ^184^ ^]^ ALKBH5 siRNA‐encapsulated lipid nanoparticles (LNPs) altered cardiac macrophage profiles and improved hypertensive cardiac fibrosis and dysfunction in mice, suggesting a potential strategy for therapy in patients.^[^ ^185^ ^]^ Double‐layer programmed drug release microneedles loaded with the METTL3 inhibitor SAH (S‐Adenosyl‐L‐homocysteine) effectively enhanced cardiac function, reduced infarct size, and mitigated cardiomyocyte death by decreasing mitochondrial fragmentation and inhibiting myofibrillar transformation in a rat myocardial infarction model.^[^ ^186^ ^]^

The m6A modification in liver fibrosis involves complex mechanisms, with multiple cellular and molecular intervention targets. METTL3 was reduced in mouse hepatocytes stimulated by free fatty acids, worsening cell steatosis by downregulating CYP4F40. This effect could be countered by cinnamaldehyde (a natural compound from *Ramulus cinnamomi*), which promotes METTL3 expression and improves steatosis.^[^ ^187^ ^]^ Resveratrol (a natural phytoestrogen) alleviated liver lipid metabolism disorders in HFD mice through changes in m6A levels, increasing the transcript levels of METTL3, ALKBH5, FTO, and YTHDF2 while decreasing YTHDF3 and m6A abundance.^[^ ^188^ ^]^ Betaine supplementation during adolescence significantly mitigated high‐fat‐induced liver function impairment by reducing FTO expression and hepatic m6A levels, thus lowering lipogenesis and enhancing lipolysis.^[^ ^189^ ^]^ Meclofenamic acid (MA), a selective FTO inhibitor, prevented triglyceride increases induced by oleic acid/dexamethasone in primary hepatocytes.^[^ ^190^ ^]^ Entacapone, another potential FTO inhibitor, affected gluconeogenesis in the liver and thermogenesis in adipose tissues via an FTO‐FOXO1 regulatory axis. It also lowered body weight and fasting blood glucose in obese mice, indicating its potential role in metabolism regulation.^[^ ^191^ ^]^

Renal fibrosis is a secondary effect of kidney injury, so the treatment based upon m6A modification focused more on improving early kidney injury. AAV9‐packaged METTL3 knockdown plasmid injected into the renal pelvis of mice reduced kidney injury and inflammation in cisplatin‐ and LPS‐induced AKI models,^[^ ^147^ ^]^ as well as decreased podocyte loss in STZ‐induced DKD.^[^ ^168^ ^]^ Furthermore, cholesterol‐conjugated YTHDF1 siRNA administered via tail vein injection reduced renal fibrosis progression due to TGF‐β treatment in the UUO mouse model.^[^ ^164^ ^]^ Genistein (a soy isoflavone) improved renal fibrosis by restoring ALKBH5 to regulate EMT, highlighting the role of m6A modification in CKD.^[^ ^159^ ^]^ Cpd‐564 as an inhibitor of METTL3 inhibited TAB3 expression and inflammatory responses while providing renoprotective effects in a cisplatin‐induced AKI mouse model.^[^ ^147^ ^]^ Additionally, TFA, a compound from *Abelmoschus manihot*, ameliorated high‐glucose‐induced pyroptosis and podocyte injury by targeting METTL3‐dependent m6A modification through NLRP3‐inflammasome activation regulation and PTEN/PI3K/Akt signaling pathways.^[^ ^192^ ^]^

## 8 Summary

In recent years, with the advancement and widespread application of high‐throughput sequencing technology, there has been extensive research on the role of m6A modifications in various diseases. Specifically, this review systematically summarizes recent research on m6A modification in fibrotic diseases affecting visceral organs such as the lung, heart, liver, and kidney. Both basic and mechanistic studies have elucidated the signaling pathways involved in m6A modifications in fibrosis and have identified potential specific targets for future clinical treatments. Furthermore, bioinformatics analysis not only provides direction for further research but also plays an increasingly important role in predicting clinical prognosis and other aspects. These studies have laid the groundwork for future exploration of novel and effective treatments. These studies have provided the groundwork for future exploration in search of innovative and effective treatments.

## Conflict of Interest

The authors declare no conflict of interest.

## Author Contributions

**Xiaoniu Dai**: Writing—original draft (lead); Writing—review & editing (lead). **Yusi Cheng**: Writing—original draft (supporting). **Wei Luo**: Writing—original draft (supporting). **Jing Wang**: Writing—original draft (supporting). **Cuifen Wang**: Writing—original draft (supporting). **Xinxin Zhang**: Writing—original draft (supporting). **Wei Zhang**: Writing—original draft (supporting). **Jie Chao**: Funding acquisition (lead); Investigation (lead); Writing—review & editing (lead).
